# Introduction to the Proteomic Analysis of Placentas with Fetal Growth Restriction and Impaired Lipid Metabolism

**DOI:** 10.3390/metabo14110632

**Published:** 2024-11-16

**Authors:** Malwina Sypiańska, Aleksandra Stupak

**Affiliations:** Department of Obstetrics and Pathology of Pregnancy, Medical University of Lublin, Clinical University Hospital n1, Staszica 16, 20-081 Lublin, Poland; molipp@wp.pl

**Keywords:** fetal growth restriction, placenta, proteome, lipoproteins, lipid metabolism

## Abstract

Fetal growth restriction (FGR) is a disorder defined as the failure of a fetus to achieve its full biological development potential due to decreased placental function, which can be attributed to a range of reasons. FGR is linked to negative health outcomes during the perinatal period, including increased morbidity and mortality. Long-term health problems, such as impaired neurological and cognitive development, as well as cardiovascular and endocrine diseases, have also been found in adulthood. Aspirin administered prophylactically to high-risk women can effectively prevent FGR. FGR pregnancy care comprises several steps, including the weekly assessment of several blood vessels using Doppler measurements, amniotic fluid index (AFI), estimated fetal weight (EFW), cardiotocography (CTG), as well as delivery by 37 weeks. Pregnancy is a complex condition characterized by metabolic adjustments that guarantee a consistent provision of vital metabolites allowing the fetus to grow and develop. The lipoprotein lipid physiology during pregnancy has significant consequences for both the fetus and baby, and for the mother. In the course of a typical pregnancy, cholesterol levels increase by roughly 50%, LDL-C (low-density lipoprotein cholesterol) levels by 30–40%, HDL-C by 25% (high-density lipoprotein cholesterol). Typically, there is also a 2- to 3-fold increase in triglycerides. Low maternal blood cholesterol levels during pregnancy are linked to a decrease in birth weight and an increased occurrence of microcephaly. FGR impacts the placenta during pregnancy, resulting in alterations in lipid metabolism. Research has been undertaken to distinguish variations in protein expression between normal placentas and those impacted by FGR. This can aid in comprehending the fundamental pathogenic mechanisms of FGR and perhaps pave the way for the creation of novel diagnostic and treatment methods. Commonly employed approaches for detecting and analyzing variations in placental proteomes include mass spectrometry, bioinformatic analysis, and various proteomic techniques.

## 1. Introduction

Fetal growth restriction (FGR) occurs when the fetus fails to achieve its full biological growth potential due to numerous pathological etiologies, including placental dysfunction. Long-term effects of FGR include perinatal morbidity and mortality, and long-term health problems, such as impaired neurological and cognitive development; it can also be a contributing factor for adult cardiovascular and endocrine diseases [1].

The Delphi FGR Criteria encompass two solitary parameters (AC-abdominal circumference or EFW-estimated fetal weight < 3rd centile Hadlock’s standard), and four contributory parameters (AC or EFW < 10th centile, AC or EFW crossing centiles by more than two quartiles on growth charts and cerebroplacental ratio < 5th centile or umbilical artery pulsatility index > 95th centile) without any congenital abnormalities being present. We can divide FGR into two types: early onset FGR (diagnosed at or below 32 weeks) and late-onset FGR (at or after 32 weeks). Early FGR can be diagnosed based on one solitary parameter or a combination of AC or EFW < 10th centile, together with another contributory parameter. One solitary parameter or at least two out of three contributory parameters are required to diagnose late-onset FGR [2].

Small for gestational age (SGA) and FGR are not the same conditions. SGA is typically defined by an estimated birth weight of less than 10th centile or a birth weight deviation of 1 or 2 standard deviations below the mean for their age, sex, and parity. There is extensive evidence showing that birth weight standards adjusted to specific populations and encompassing ethnicity and the height and body mass index of the mother can lead to the more efficient diagnosis of FGR [3].

### 1.1. Pathogenesis and Etiology

The most common underlying pathogenesis of FGR involves the abnormal development and function of the placenta, which leads to reduced nutrient and oxygen supply. Due to the failure of extravillous cytotrophoblast invasion, placental structure is poor, and spiral arteries are inadequately remodeled, which impairs perfusion. This results in altered vascular resistance, intra-placental vascular lesions, and reduced surface area for the exchange between the mother and the fetus [3].

Preeclampsia and gestational hypertension, poor maternal cardiovascular adaptation to pregnancy, multifetal gestation, maternal diabetes, maternal environmental influences, such asthma and stress, lifestyle factors, including smoking, drug use, and malnutrition may lead to dysfunctional placental development and function. Genetic and otherwise fetal abnormalities and infections, for example cytomegalovirus, syphilis, hepatitis C and SARS-CoV-2, can also be associated with FGR [3]. The study conducted at The Clinic for Gynecology and Obstetrics at the University Clinical Centre, Kragujevac, Serbia, included 320 pregnant women and revealed the correlation between FGR during the second and third trimesters and lower body height and proteinuria. Shorter women with proteinuria, using corticosteroids or smoking during pregnancy, more commonly gave birth to SGA babies [4].

The Polish Society of Gynecologists and Obstetricians’ recommendations on the diagnosis and management of fetal growth restriction divided risk factors of FGR into small and large risk factors [5]. At least one large factor or three small risk factors identified allowed for the diagnosis of the elevated risk of FGR. Risk factors can be maternal, paternal, and associated with obstetrician history and current pregnancy (see Table 1 and Table 2).

FGR fetuses are classified into four stages:

Stage I—EFW < 3rd centile or CPR (cerebro-placental perfusion) < 5th centile or MCA pulsatility index < 5th centile (both persisting 12 h apart) or mean UtA (uterine artery) pulsatility index > 95th centime (polish recommendations add AC < 3rd centile).

Stage 2—AEDV (absent enddiastolic velocity) or AoI (aorta isthmus) revered diastolic velocities—both persisting 12 h apart (in Polish Recommendations only AEDV).

DV (ductus venosus) pulsatility index > 95th centile or REDV (reverse enddiastoloc velocity)—both persisting 12 h apart.

DV absent/reversed EDV persisting 12 h apart or pathological CTG (cardiotocography) [6].

The Polish Recommendations for the diagnosis of preeclampsia increases stage of FGR by one [1].

### 1.2. Prevention

**Figure 1:**
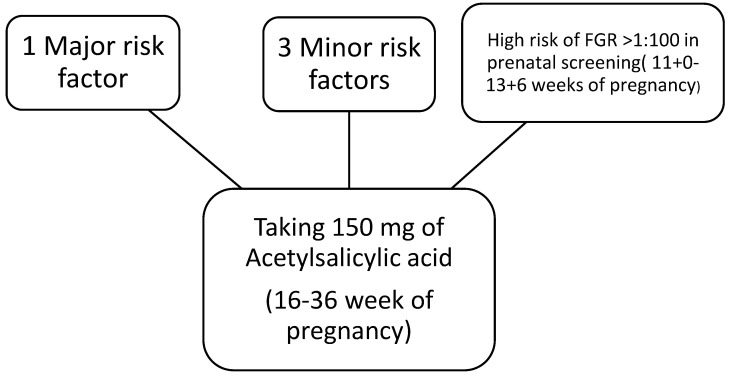
Indications for taking acetylsalicylic acid during pregnancy.

Randomized clinical trials have shown that using aspirin prophylactically in high risk women can reduce the risk of fetal growth restriction. A high risk of FGR can be diagnosed when one large or three small risk factors of FGR are present. A high risk of FGR can be recognized using prenatal screening between the 11th and 13th weeks of gestation using the Doppler assessment of blood flow in the UtA, or an assessment of the mean arterial pressure and the level of the placental growth factor PlGF in the maternal blood (Figure 1). It is recommended to take 150 mg of acetylsalicid acid before the 16th week of pregnancy and continue until the 36th week in pregnant women with a high risk of FGR [1].

The use of aspirin has been proven to prevent fetal growth restriction in pregnancy. It is related to a dose–response effect. Taking low doses of aspirin at >16 weeks’ gestation has a minor or no impact on the risk of FGR. Women with a high risk of FGR should be diagnosed in early pregnancy [7].

### 1.3. Management

Flowchart of management depends on the degree of fetal growth restriction (Table 3). The Polish Recommendations concerning the diagnosis and management of fetal growth restriction are similar to the Barcelona Protocol, but there are a few differences. Stage I of FGR encompasses a weekly multivessel Doppler measure and AFI, the measurement of EFW every two weeks, CTG with short term variability every week after 34 weeks of pregnancy, and delivery by 37 weeks. In stage II, it is obligatory to repeat the multivessel Doppler every two days, consider steroids if age of gestation is below 34 weeks, intensified CTG vision, and delivery by 34 weeks. In stage III of FGR we have to repeat the multivessel Doppler every 12–24 h and perform CTG. In the case of incorrect CTG or persistent AEDV, delivery by caesarean section after steroids is recommended. In stage IV, continuous CTG is mandatory, steroids should be given, and the caesarean section should be performed immediately. Short-term variability < 3.5 in CTG longer than 40 min, or repeated decelerations, are indications for delivery [1]. 

## 2. Metabolic Adaptations in Pregnancy

**Figure 2:**
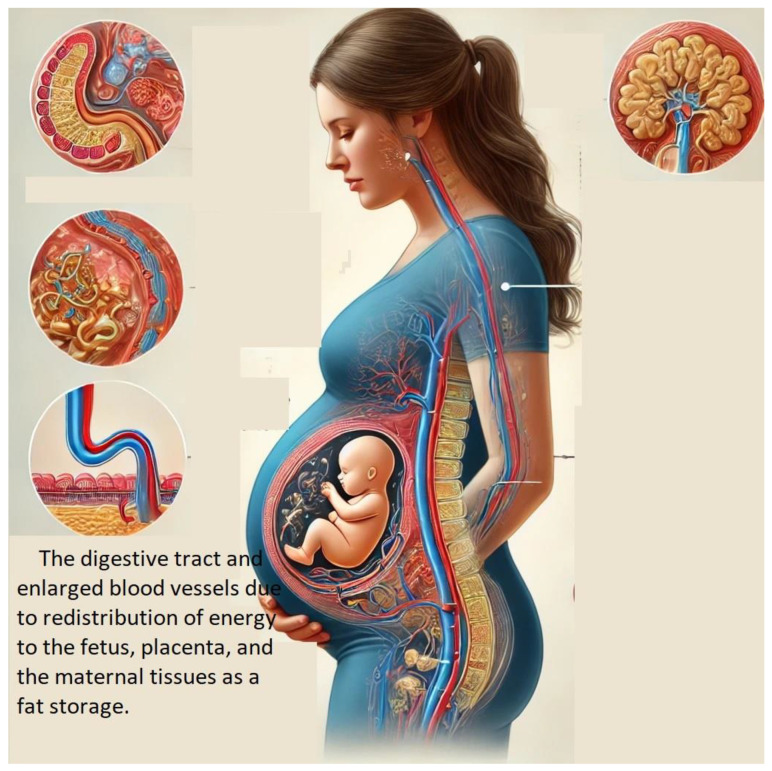
The physiological changes in a woman’s body during the pregnancy.

In the course of pregnancy, multiple adaptations occur. They play an important role in the continuous supply of necessary metabolites essential for fetal growth and development. Metabolic adaptations are important in the course of pregnancy (Figure 2). 

**Figure 3:**
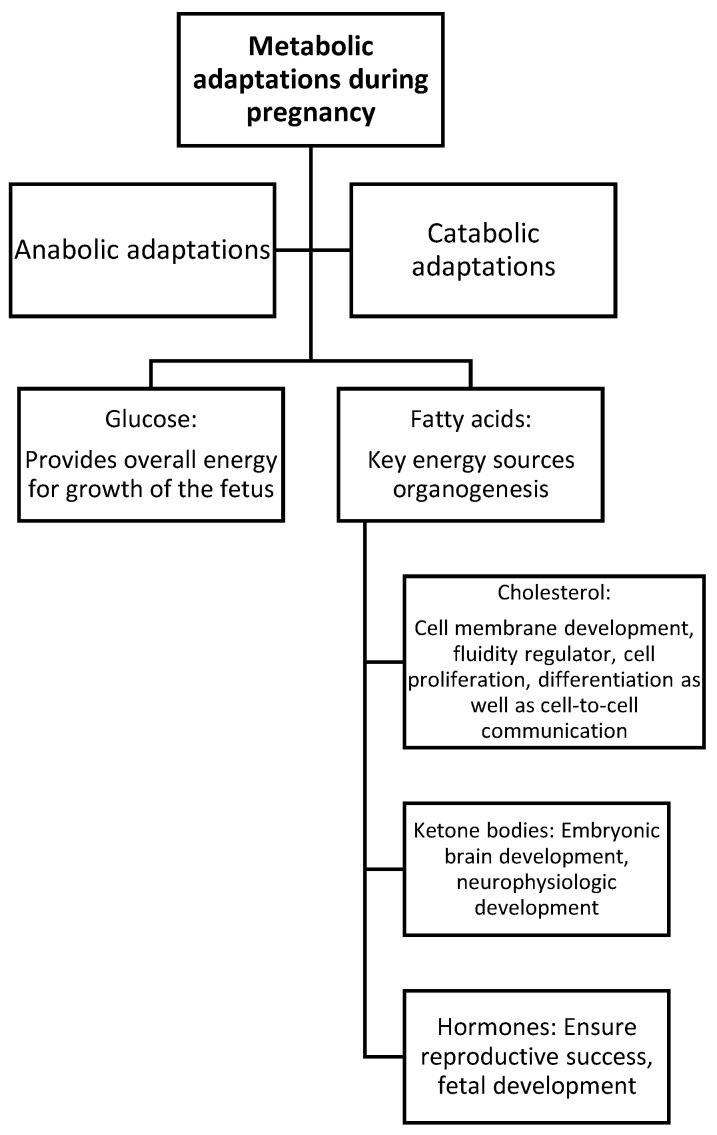
Overview of metabolic adaptions during pregnancy [8].

The adaptations ensure that the mother stores enough energy to meet the demands of pregnancy. They also help to prepare for lactation and support proper fetal growth in the womb [8]. Lipoprotein physiology during gestation significantly influences the development of a fetus and newborn. It also has implications for the mother (Figure 3). Fetal development is closely related to cholesterol and fatty acids. The occurrence of physiological changes in the course of gestation contribute to the change in lipid profiles of healthy pregnant women. During early pregnancy, body fat accumulation increases, which is associated with hyperphagia and enhanced lipogenesis [9]. The increased insulin sensitivity and production of progesterone, cortisol, leptin, and prolactin are a key factor for increased fat storage [10]. During late pregnancy, fat deposits break down faster, ensuring the proper development of the fetus [9]. Maternal lipid metabolism consists of anabolic and catabolic phases. In the first and second trimesters, an anabolic phase with an elevated deposition of lipids in maternal tissues occurs and helps to prepare the mother’s organism for the increases in fetal energy needs in late pregnancy. The catabolic phase occurs during the third trimester, in which fat deposits break down faster due to increased adipose tissue lipolytic activity. Enhanced lipolysis of stored triglycerides in adipocytes causes insulin sensitivity to decrease. Lipolysis in adipocytes is stimulated by the elevated human placental lactogen. The catabolism increases primary substrates for the developing fetus [8,10].

In normal pregnancy there is a total cholesterol level increase of approximately 50%, an LDL-C increase by 30–40%, an HDL-C by 25%, and triglycerides by 2- to 3-fold. The three-cohort study conducted in Finland reassessing the metabolic profiles of pregnant and non-pregnant women revealed that all lipoprotein subclasses and lipids had been significantly elevated in pregnant women in comparison to non-pregnant women. Lipoprotein and triglyceride concentrations differed the most, and many fatty acids and amino acids were also significantly different. The impact of pregnancy is exceptionally large for metabolism, which gradually increases across the trimesters to generally normalize within 3–6 months postpartum. 

### 2.1. Lipids in Fetal Development

Lipids play a crucial role in fetal growth and development. In normal pregnancy, lipid levels increase. Cholesterol is crucial for the formation of cell membranes [10]. It determines membrane fluidity and passive permeability. It also has a great impact on cell proliferation, differentiation, and cell-to-cell communication. Different metabolic processes are regulated by cholesterol and its oxidative derivatives. Cholesterol is important for embryonic and fetal development. Studies have revealed that, for each kilo of tissue that is added to the body of a growing fetus, 1.5–2.0 g of cholesterol is needed [8]. The fetus may obtain cholesterol from endogenous synthesis as well as from the yolk sac and the placenta. Cholesterol derived from the mother is important for fetal growth and development. Low maternal serum cholesterol levels during gestation contribute to reduced birth weight and microcephaly. Gestational hypercholesterolemia promotes early atherogenicity [8].

Exogenous cholesterol must be transported across the tissues separating the mother and fetus to make it available to the fetus. Cholesterol is taken up on trophoblasts’ apical or maternal side via receptor-mediated and receptor-independent transport processes. Apolipoprotein lipids are then transported across cellular barriers and delivered into the fetal circulation on the basolateral or fetal side of trophoblasts. There is an ongoing intense study of how placental endothelial cells transport and deliver substantial cholesterol amounts to the microcirculation of the fetus. The efflux of cholesterol regulation has been closely studied [10].

Apolipoprotein B100, an effective biomarker for distinguishing between intrauterine growth restriction and control groups, serves as the principal structural protein in atherogenic lipoproteins [11]. It reflects the total amount of potentially atherogenic circulating lipoproteins, such as low-density lipoprotein (LDL), intermediate-density lipoprotein (IDL), very low-density lipoprotein (VLDL), and lipoprotein (a). Chylomicrons containing apolipoprotein B100 can transport large quantities of cholesterol and triglycerides, as well as important lipids such as lipophilic vitamins and glycolipids. The critical role of apolipoprotein B-containing lipoprotein secretion is particularly pronounced in the liver and intestine, where protein necessary for the export of significant amounts of lipids to peripheral tissues is transferred by both apolipoprotein B and microsomal triglyceride. There are two primary isoforms of apolipoprotein B: apolipoprotein B100 and apolipoprotein B48. Apolipoprotein B100 is synthesized in the liver and represents the circulating apo B particles. Additionally, the placenta can secrete apo B particles. Notably, this study found that low apo B levels may indicate placental dysfunction in many of IUGR cases. Therefore, apo B levels could directly indicate placental pathology and may be instrumental in evaluating the pathophysiology of individual patients. The potential of apolipoprotein B and apolipoprotein A1 have been examined as markers of lipoprotein metabolism. Apolipoprotein A1, the main structural protein HDL particles, has diverse biological functions, one of which are anti-inflammatory properties. They encompass the inhibition of LDL oxidation and the removing of toxic phospholipids. In other studies (apo B-containing) LDL particles, as indicated by LDL-cholesterol levels, remain unaltered in preeclampsia (PE) unless significant placental dysfunction is present, which is indicated by the presence of FGR [12]. This observation aligns with a study that found unchanged apo B levels in PE but reduced apo A1 levels [13]. Consequently, the apo B/apo A1 ratios were elevated in PE.

It is widely considered that maternal triglycerides constitute a crucial nutrient for fetal growth. Higher maternal triglyceride concentration contributes to larger neonatal fat mass and higher birth weight, which has been revealed in numerous observational studies [14].

Fatty acids are essential for the developing fetus to assist rapid cellular growth and activity [14]. They are a key energy source for proper fetal development and function.

A study conducted as a part of larger prospective research program on FGR at the Department of Maternal-Fetal Medicine in Hospital Clinic in Barcelona has proven lower plasma concentrations of cholesterol-intermediate density lipoprotein (IDL), triglycerides-IDL, and high-density lipoprotein (HDL) in the FRG group in comparison to the control group. On the other hand, growth-restricted fetuses showed considerably higher plasma concentrations of cholesterol and triglycerides transporting lipoproteins (LDL, IDL and VLDL) [15].

Research conducted at Department of Obstetrics and Neonatology in a teaching hospital in North India investigated the lipid profile of woman with and without fetal growth restriction. The pregnant women in the FGR group had lower total cholesterol, triglycerides, LDL, HDL, TC/HDL, and LDL/HDL ratios in comparison to the control group [16].

A prospective cross-sectional case-control study was conducted to investigate lipid levels of mothers and fetuses. Enzymatic analysis and immune-turbidimetric enzymatic assays were used [17]. The results of this study show that ApoA1 and ApoB levels did not differ between clinical groups in the maternal circulation, however, important variations were observed in the fetal circulation. ApoB levels were increased in preeclampsia (PE), fetal growth restriction (FGR), and PE + FGR in comparison to normal pregnancies. Elevated ApoB levels, as well as a higher ApoB/ApoA1 ratio, have been identified as predictive factors for atherogenesis in later life. Fetal growth restriction and poor growth in the early years of life have been found to be a probable cause of cardiovascular diseases in adulthood.

In the control observational study conducted by Sydney Medical School, triglyceride levels were increased in preeclampsia and cord blood fetal growth restriction with preeclampsia groups compared to normal pregnancies. Total cholesterol, high density cholesterol, and low density cholesterol did not show any important gestational variation between four clinical groups (normal pregnancies, pregnancies with preeclampsia, pregnancies with fetal growth restriction, and pregnancies with preeclampsia and fetal growth restriction) in the maternal or fetal circulation [17].

Another research population-based cohort in Finland showed that the most significant metabolic changes during pregnancy were observed in the circulating concentrations of IDL, LDL, HDL, and triglycerides. The increase in triglycerides was more impressive than the increase in phospholipids and cholesterol. The results of the same study revealed that total circulating maternal fatty acid concentration steadily increased during gestation with the overall increase in lipoproteins [14].

Maternal lipid concentration is associated with fetal growth restriction not only in the second and third trimesters of pregnancy. The population-based prospective cohort study conducted at the University Medical Centre in Rotterdam showed that higher triglyceride and cholesterol concentrations in early pregnancy are associated with increased embryonic size, especially in overweight women. Maternal lipid concentrations may be a marker of early embryonic and fetal growth [18].

While much attention has been given to understanding the specific metabolic alterations in obese pregnant women, likewise the preconception period plays an important role in fetal programing [19]. Pregestational obesity does not significantly impact the fundamental parameters of carbohydrate metabolism in pregnant women, but it does disrupt the lipid profile, characterized by a notable rise in triglyceride levels and a reduction in HDL cholesterol levels in the serum [20]. Additionally, pre-existing obesity leads to elevated levels of leptin and resistin in the serum of pregnant women, likely due to the increased amount of adipose tissue.

### 2.2. Proteomic Analysis

New techniques have been examined to extend the spectrum of screening biomarkers useful in detecting pregnancy-related problems, including FGR. LC-MS is a powerful analytical method used to detect and analyze various chemical compounds. The advantage of this method is high accuracy and sensitivity [11].

Miao et al. conducted one of the first studies to identify proteins differentially expressed in normal and FGR placentas [21]. They identified 1198 proteins, which were analyzed using the Thermo-fisher Q-Exactive Orbitrap. Mass spectrometry data obtained with Mascot (version 2.3.01) were analyzed, and 95 proteins showed significant (*p* < 0.05) differential expression between the normal and FGR placentas. They were associated with two primary molecular networks related to erythropoiesis and oxidative stress. Numerous proteins, including NADPH oxidase, low-density lipoprotein (LDL), and SERPINA1, are crucial for the development of FGR. NADPH oxidase is an enzyme complex capable of producing substantial amounts of reactive oxygen species (ROS).

In a recent study of mother blood plasma proteomics, thirteen potential markers of FGR, including Gelsolin, Alpha-2-macroglobulin, Apolipoprotein A-IV, Apolipoprotein B-100, Apolipoprotein(a), Adiponectin, Complement C5, Apolipoprotein D, Alpha-1B-glycoprotein, Serum albumin, Fibronectin, Glutathione peroxidase 3, and Lipopolysaccharide-binding protein, were found to be interconnected in a protein–protein interaction network [22]. These proteins act in plasma lipoprotein assembly, remodeling, and clearance; lipid metabolism, particularly involving cholesterol and phospholipids; hemostasis, including platelet degranulation; and immune system regulation. Moreover, 18 proteins were identified as specific to either early or late-onset FGR, with distinct patterns observed in coagulation and fibrinolysis systems between the two types.

The maternal proteomic profiling (2D nano LC-MS/MS proteomics analysis) revealed significant changes in 25 proteins in late-onset FGR [23]. The direct protein–protein interaction network indicated that Neurogenic locus notch homolog protein 1 (NOTCH1) emerged as the most significant potential upstream regulator of the observed protein profile. Gene ontology analysis of these proteins identified their involvement in 14 canonical pathways. The authors conclude that potential therapeutic strategies for late-onset FGR could involve modulating key proteins and pathways involved in placental function, oxidative stress, lipid metabolism, and immune regulation, using pharmacological, gene-based, or nutritional approaches.

In the study first conducted by the members of our team in 2022, chromatographic mass spectrometry was utilized to compare the placental proteome profiles between pregnancies affected by late-onset FGR and those with normal pregnancies [24]. The analysis identified 356 different proteins involved in regulating gene transcription, inhibiting proteolytic enzymes, controlling trophoblast proliferation and angiogenesis, and mediating inflammatory responses. In the placental proteome of FGR cases, other detected proteins were primarily associated with oxidative stress responses, cellular oxidation and detoxification, apoptosis, hemostasis, catabolic processes, energy transduction, cell proliferation, differentiation, and intracellular signaling. Therapeutic approaches could focus on proteins and signaling pathways identified as significant in late-onset FGR, such as those involved in placental function, nutrient transport, or vascularization.

## 3. Conclusions

Not many studies have been conducted comparing the placental proteome in normal pregnancies to those with FGR. These studies aim to identify differences in protein expression between healthy placentas and those affected by FGR, which can help with understanding the underlying pathological mechanisms of FGR and potentially contribute to developing of new diagnostic and therapeutic methods. Techniques, including mass spectrometry, bioinformatic analysis, and various proteomic methods are commonly used in these studies to detect and analyze differences in placental proteomes. Studies on lipid metabolism in the placenta in the context of normal pregnancy and FGR are also extensively researched. These studies focus on understanding how changes in placental lipid metabolism may contribute to the onset of FGR, and what differences exist in the expression of proteins and metabolic enzymes between healthy placentas and those affected by FGR. Techniques such as liquid chromatography-mass spectrometry (LC-MS) and various enzymatic assays are used in the identification and quantification of lipids and metabolic compounds in the placenta. Such research can not only provide an alternative perspective to better understand the role of lipids in FGR development, but also identify potential therapeutic targets related to lipid metabolism regulation during pregnancy.

## Figures and Tables

**Table 1:** Major risk factors for FGR [1].

Maternal	Paternal	Current Pregnancy	Obstetric History
Antipholipid syndrome Diabetes-related angiopathy Renal failure Intense physical activity >40 years old Cocaine Maternal birth weight < 10th centile Chronic hypertension Smoking > 10 cigarettes/day	Paternal birth weight < 10th centile	Preeclampsia Threatened abortion with heavy bleeding Pregnancy-induced hypertension-severe Hyperechogenic fetal intestine in II trimester on ultrasound	Previous neonate with birth weight < 10th centile

**Table 2:** Minor risk factors for FGR [1].

Maternal	Current Pregnancy	Obstetric History
Maternal first pregnancy Fruit low intake prior to pregnancy IVF BMI ≥ 30 (Obesity) BMI < 20 (Underweight) BMI 25–29.9 (Overweight Maternal age > 35 years	Caffeine intake ≥ 300 mg/daily in the III trimester Mild gestational hypertension	Mild preeclampsia Time between pregnancies ≥ 60 months Time between pregnancies < 6 months

**Table 3:** Management protocol in FGR [8].

SGA	FGR Stage I	FGR Stage II	FGR Stage III	FGR Stage IV
EFW > 3rd centile Normal multivessel Doppler findings	EFW < 3rd centile or EFW > 3rd centile Umbilical artery PI > 95thcentile CPR < 5th centile UtA PI > 95th centile	Umbilical artery Absent end-diastolic flow	Umbilical artery reversal in end-diastolic flow Ductus venosus PI > 95th centile	Ductus venosus absent or reversal of a wave
Repeat US every 2 weeks Assess biometry, AFI, multivessel Doppler Delivery by 40 weeks	Weekly multivessel Doppler EFW+AFI+Multivessel Doppler every week Maternal evaluation of hypertension	Repeat multivessel Doppler every 2 days Consider steroids if GA < 34 weeks Delivery by caesarean section by 34 weeks	Repeat multivessel Doppler every day Consider MgSO_4_ for neuroprotection if GA < 32 weeks Consider steroids if GA < 34 weeks Delivery by caesarean section by 30 weeks	Repeat multivessel Doppler every 12 h Delivery bycaesarean section by 26 weeks

Delivery is indicated in all cases: abnormal NST-Oligohydroamnios (AFI < 2 cm). Presence of uncontrolled maternal hypertensive disorders or any other maternal condition that warrants delivery.

## Data Availability

No new data were created or analyzed in this study. Data sharing is not applicable to this article.

## Abbreviations

AC	abdominal circumference
AEDV	absent end diastolic velocity
AFI	amniotic fluid index
Apo-1	apolipoprotein-1
BMI	body mass index
CPR	cerebro-placental-ratio
CTG	cardiotocography
EFW	estimated fetal weight
FGR	fetal growth restriction
GA	gestational age
HDL	high density lipoproteins
IVF	in vitro fertilization
MS	mass spectrometry
LC	liquid chromatography
LDL	low-density lipoprotein
PE	preeclampsia
PI	pulsatility index
PlGF	placental growth factor
REDV	reversed end diastolic velocity
ROS	reactive oxygen species
SGA	small for gestational age
UtA	uterine artery
VLDL	very low-density lipoprotein
